# Hydrodynamic Properties of Polymers Screening the Electrokinetic Flow: Insights from a Computational Study

**DOI:** 10.3390/polym11061038

**Published:** 2019-06-11

**Authors:** Peng Wu, Tao Sun, Xikai Jiang, Svyatoslav Kondrat

**Affiliations:** 1 Department of Energy and Power Engineering, Huazhong University of Science and Technology, Wuhan 430074, China; 2 China-EU Institute of Clean and Renewable Energy, Huazhong University of Science and Technology, Wuhan 430074, China; total_sun@hust.edu.cn; 3 State Key Laboratory of Nonlinear Mechanics, Institute of Mechanics, Chinese Academy of Sciences, Beijing 100190, China; xikaij@imech.ac.cn; 4 Department of Complex Systems, Institute of Physical Chemistry, PAS, 01-224 Warsaw, Poland; svyatoslav.kondrat@gmail.com or skondrat@ichf.edu.pl

**Keywords:** polymeric coatings, Stokes radius, hydrodynamic shielding, molecular dynamics simulations, Navier-Stokes-Brinkman model

## Abstract

Understanding the hydrodynamic properties of polymeric coatings is crucial for the rational design of molecular transport involving polymeric surfaces and is relevant to drug delivery, sieving, molecular separations, etc. It has been found that the hydrodynamic radius of a polymer segment is an order of magnitude smaller than its physical size, but the origin of this effect does not seem to be well understood. Herein, we study the hydrodynamic properties of polymeric coatings by using molecular dynamics simulations, navigated by the continuous Navier-Stokes-Brinkman model. We confirm that the averaged hydrodynamic radius of a polymer bead is about one order of magnitude smaller than its physical radius, and, in addition, we show that it exhibits a strong dependence on the degree of polymerization. We relate this variation of the hydrodynamic radius to the structural properties and hydrodynamic shielding by surrounding polymer beads. This is done by separating the effects originating from near and far beads. For the near beads, shielding is mainly due to the two nearest beads (of the same polymer) and leads to about a 5-fold reduction in the hydrodynamic radius. Assuming the additivity of the hydrodynamic shielding by far beads, we suggest a simple model, which captures correctly the qualitative behaviour of the hydrodynamic radius with the degree of polymerization. The revealed shielding effects provide important insights relevant to the advanced modelling of hydrodynamic properties of polymeric coatings.

## 1. Introduction

Grafting polymers on surfaces is a versatile way to tune their physicochemical properties, which has numerous applications [1,2,3]. For instance, grafting nanocarriers with polymers improves their bio-compatibility and stability, making them a promising candidate for drug transport [4,5]. Channels coated with polymers regulate flows and enable “smart” functionalities in sieving, separation, and remote control [6,7,8]. Such wide-spread applications have stimulated the fundamental research on transport phenomena involving polymer-grafted surfaces [9,10,11,12,13]. Indeed, electrokinetic transport is widely used in micro/nanoscale systems because of its ease-to-use scale-up capabilities [14,15]. Typical examples include electrophoresis of particles [16], where particles are in motion relative to stationary fluids, and electroosmotic flows (EOFs) in nanochannels [17], where fluids are in motion relative to stationary walls. The fundamental transport model governing these two phenomena is the same, and hence the insights obtained from one phenomenon can be applied to the other [6].

Electrokinetic transport of hard-core particles is relatively well understood now [18,19], but understanding the transport involving polymer-coated surfaces remains a challenge due to the complexity of hydrodynamic interactions [20]. Frequently, polymeric coatings are modeled as porous media exerting a drag force on their environment [21,22]. The hydrodynamics are then characterized by the friction coefficient of polymers [23] or by the hydrodynamic radius of resistance segments [24]. For instance, polymeric coatings have been modeled as a distribution of resistance centers described by certain distribution profiles such as a uniform layer [23], a diffusive layer [20], a Gaussian-like layer [24], and a self-consistent polymer profile [25]. Harden et al. [26] studied the relation between the electroosmotic mobility of the polymer-grafted surfaces and the conformation of polymers. The hydrodynamic properties of polymeric coatings were examined by a mean-field approach, in which again the distribution of polymer beads was approximated by a certain profile of resistance centers. However, such a mean-field approach neglects the molecular nature of polymer chains, i.e., that the polymers are chains of connected beads rather than isolated resistance centers.

To model more accurately the hydrodynamic properties of polymers, it is necessary to consider the hydrodynamic interactions at nanoscale [27,28]. This can be achieved by molecular simulations [29], which allow one to take into account such interactions explicitly and offer important molecular insights. For instance, Grest [30] studied the interfacial sliding of polymeric coatings using molecular dynamics (MD) simulation, and Gama Goicochea et al. [31] calculated the friction coefficient of surfaces functionalized with polymer chains using dissipative particle dynamics. Sarkar et al. [32] investigated the structure and hydrodynamic properties of nanocarriers under flow conditions by Brownian dynamics simulations. They found that the conformations and orientations of nanocarriers under shear depend on the applied shear rate and crosslinking density of nanocarriers. Hill [25] interpreted the electrophoretic mobility data of PEG coated liposomes [33] by full electrokinetic model and achieved an excellent agreement between the theory and experiment, but the fitted hydrodynamic radius of a polymer segment turned out to be unusually small. More recently, Raafatnia et al. [34] studied electrophoresis of soft particles by Lattice-Boltzmann (LB) simulations. The LB results were matched with the predictions of the continuous Darcy-Brinkman equation, in which the Stokes radius of a polymer segment was obtained from the LB simulations and used as an input.

The aforementioned studies of polymeric coatings on macro- and microscopic levels have unveiled some of the most important hydrodynamic properties. However, several key questions remain unanswered: Why the Stokes radius of the polymer bead in a polymeric coating, $a_{\mathrm{bead}}$, is much smaller than its physical radius? Whether and to what extent the conformation of polymers affects $a_{\mathrm{bead}}$? It is vital to resolve these issues in order to model more accurately the hydrodynamic and rheological properties of polymeric coatings, which has been identified as a key challenge in the electrokinetic transport in polymer-coated systems [3]. In this work, we investigate polymeric coatings, screening an EOF, by MD simulations and by continuous, one-dimensional Navier-Stokes-Brinkmann (NSB) model. We extract the effective Stokes radius of polymer beads, by matching the simulation results with the results of the NSB model, and demonstrate that $a_{\mathrm{bead}}$ varies significantly with the chain length *N* of polymers. We rationalize the reduction of $a_{\mathrm{bead}}$, and its variation with *N*, by relating the structural and hydrodynamic properties of polymer’s beads in coatings.

## 2. Methods

Electro-osmotic flow (EOF) in a polymer-coated nanoslit was modeled by using molecular dynamics (MD) simulations and the continuous Navier-Stokes-Brinkman (NSB) model. The MD simulations were used to calculate the velocity profile of the EOF across the slit (as well as the densities of the solvent, ions and polymers’ beads). The NSB equation was applied to obtain the flow field across the slit and to infer the Stokes radius of the polymer’s bead, $a_{\mathrm{bead}}$, by matching the flow velocity with that obtained by MD simulations.

### 2.1. MD Simulation

**Figure 1:**
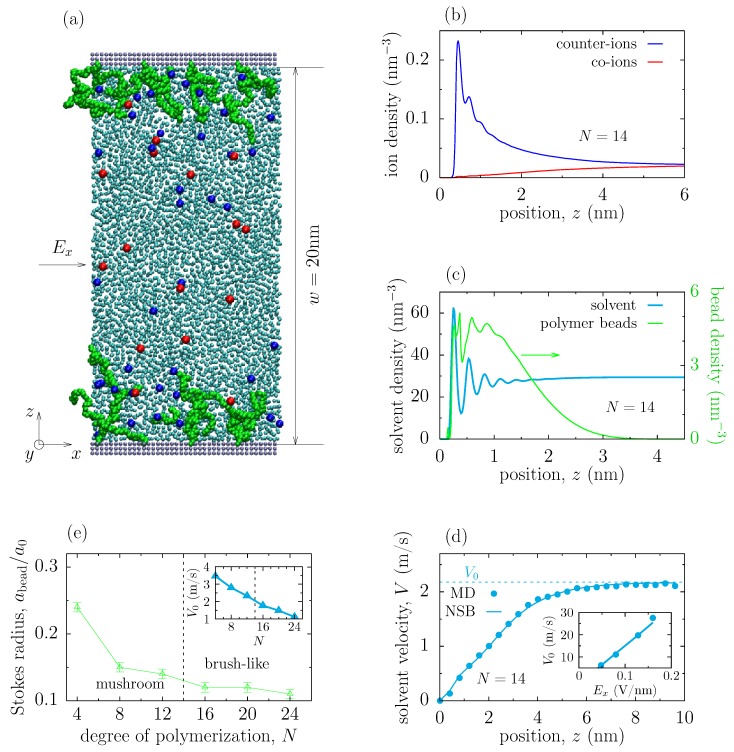
Polymer coating screening the electrokinetic flow in a slit. (**a**) Snapshot from molecular dynamics (MD) simulations. A homogeneous electric field $E_{x}$ is applied in the *x* direction to drive the electro-osmotic flow, which is screened by the polymeric coatings attached to the slit walls. The ions are depicted by red and blue colors, and the solvent is turquoise; (**b**,**c**) Averaged ion density (**b**), and polymer bead and solvent densities (**c**), across the slit from MD simulations; (**d**) Average solvent velocity across the slit from MD simulations and from the continuous Navier-Stokes-Brinkmann (NSB) model. The inset shows the solvent velocity in the middle of the slit as a function of the applied field. In (**a**–**d**), the degree of polymerization N=14 so the polymers have 3N+2=44 free beads (see Section 2). The polymers are placed on a square lattice with the lattice constant a=2.5 nm on both slit walls. The slit width is w=20 nm. Effective hydrodynamic radius of the polymer bead in the NSB model $a_{\mathrm{bead}}\approx 0.019$ nm; (**e**) The effective hydrodynamic radius of the polymer bead as a function of the degree of polymerization *N*; $a_{\mathrm{bead}}$ is normalized to the Lennard-Jones radius of the bead $a_{0}=0.156$ nm. The inset shows the mid-plane solvent velocity *versus* *N*.

The MD system is shown in Figure 1a and consists of an electrolyte confined between two parallel walls grafted with polymer chains. The grafting density of polymers was 0.164 nm${}^{-2}$ as in the experiments [33]. The walls were constructed of three layers of atoms placed on the face-centered cubic lattice with atom density 33.3${\mathrm{nm}}^{-3}$. The wall atoms in contact with the electrolyte were charged, with the surface charge density $\sigma_{s}=3.28$ × $10^{-2}$ C/m${}^{-2}$, and the atoms of the other layers were left uncharged. In order to model correctly the solvation behaviour at the walls, we introduced additional “virtual” walls, placed 0.1 nm away from the innermost layers, which interact via the Lennard-Jones (LJ) interactions with the ions only; these walls shift the ion distribution to that obtained using the SPC/E model of water [35,36,37]. The size of the system in the *x* and *y* direction was 9.88 nm and the slit width was w=20nm (*w* is the distance between the innermost layers of the two walls, and z=0 was chosen to be at the center of the innermost layer of the bottom wall). A vacuum space of width 30 nm was added in the *z* direction. System parameters are summarized in Table S1. The corresponding parameter and coordinate files can be found in Supplementary Materials.

In order to focus on the hydrodynamic properties, unobscured by chemical details of real intermolecular interactions, we used the Weeks-Chandler-Andersen (WCA) interaction potential [38] between the solvent molecules and between the solvent and the ions, and charged WCA particles were used to model ions. Dielectric constant was taken $\varepsilon_{s}=78$ to account for the dielectric properties of the solvent. The ionic strength of electrolyte was 3.4 $\times 10^{-2}$ mol/L as in the experiments (0.56 M) [33]. We used the force field parameters for solvent and ions as in Ref. [39] (Table S2).

Polyethylen glycol (PEG), H−(O−CH_2_−CH_2_)n −OH, was simulated by using the united-atom model, in which every backbone atom (carbon and oxygen) is modeled as LJ spheres (beads) with the same force-field parameters, while the hydrogen atoms are ignored; also the solvent (water) was consistently modeled as a single bead with the same LJ parameters (Table S2). Thus, within our model, a monomer consisted of three beads and a polymer of degree *n* of 3n+1 beads. However, we used the first two beads of a polymer to attach it to the wall, and therefore we had N=n−1 *free* monomers and 3N+2 free beads. Force field parameters for polymers were taken from the OPLS parameters for hydrocarbon [40]. The topology files, which define bonds, bond angles and dihehedral interactions of polymers, were obtained by submitting the molecular structure of the hydrocarbon chains to PRODRG2 server [41]; the dihedral parameters were then tuned to obtain a flexibly deformed polymer. The corresponding itp files can be found in Supplementary Materials.

We chose to develop the united-atom model in order to capture the fine-grain features of PEG polymers, especially overlapping atoms. To check this model, we calculated the scaling law of the gyration radius, $R_{g}$, as a function of the degree of polymerization *N* (i.e., the exponent γ in $R_{g}∼N^{\gamma }$), and obtained γ=0.65. For a random-walk polymer, the scaling of gyration radius of the polymer chain is 0.59 [42]. This difference in γ might be due to the strong interactions between the polymer and solvent. However, the polymers in our united-atom model can freely deform, mimicking the configurations of real polymers.

MD simulations were performed by using simulation package Gromacs 4.5.1 [43]. The cut-off radius of the Lennard-Jones potential was $2^{1/6}$ σ (σ= 0.3 nm) to mimic the solution condition of polymers in good solvent [30]. Electrostatic interactions were computed by using the Particle-Mesh Ewald (PME) in 2D (in *x* and *y* dimensions) [43] with a cut-off radius 1.3 nm. To generate an EOF, we applied an electric field in the *x* direction of strength $E_{x}=1.6\times 10^{-2}$ V/nm. Temperature was 300 K and we used V-rescaling thermostat [43]; the pressure was one bar. The time step was 4 fs, and the simulations were run for 10 ns to reach a steady state, which was followed by a production run of 100 ns. In each case we performed three independent simulation runs.

### 2.2. Navier-Stokes-Brinkman Model

The Navier-Stokes-Brinkman (NSB) equation is [25]

(1) $$\begin{array}{c} \frac{d}{dz}\left[\mu \left(z\right)\frac{du_{eo}\left(z\right)}{dz}\right]-6\pi \mu \left(z\right)n\left(z\right)a_{\mathrm{bead}}K\left(\phi \left(z\right)\right)u_{eo}\left(z\right)+\sum_{i=1}^{M}Fc_{i}\left(z\right)E_{ext}=0, \end{array}$$

where μ(z) is the fluid viscosity, $u_{eo}$ is the EOF velocity, n(z) is the number density distribution of polymer beads, $a_{\mathrm{bead}}$ is the effective Stokes radius and ϕ(z) is the volume of polymer beads. The function K(ϕ) accounts for the correlations between homogeneously distributed spherical particles [44] (Section S4A in Supplementary Materials), *F* is the Faraday constant, $c_{i}\left(z\right)$ the ionic concentration of species *i*, M=2 is the number of ionic species, and $E_{ext}$ is an applied electric field. On the left hand side of Equation (1), the first term denotes the viscous force, the second term is the hydrodynamic drag exerted by a polymeric coating on the fluid, and the last term is the driving force of the EOF.

The functions n(z) and $c_{i}\left(z\right)$ were measured directly in MD simulations and used as an input in Equation (1). For the fluid viscosity, μ(z), we used the bulk solvent viscosity, combined with the Einstein relation model to account for the effect of polymer beads [45] (Figure S6, Section S4B in Supplementary Materials; for a more rigorous method to determine the fluid viscosity, see Ref. [46]). No-slip boundary condition was applied at the wall and the mirror symmetry of EOF velocity profile was imposed at the center of the slit (Section S4C in Supplementary Materials). The hydrodynamic radius $a_{\mathrm{bead}}$ was treated as an adjustable parameter to match the EOF velocity profile obtained by MD simulations.

## 3. Results and Discussion

We have considered a slit filled with an aqueous electrolyte; the slit walls were positively charged and grafted with polyethylene glycol (PEG) polymers (Figure 1a; see Section 2 for modelling details). The electro-osmotic flow (EOF) was induced by applying a homogeneous static electric field $E_{x}$ in the *x*-direction along the slit. From molecular dynamics (MD) simulations, we obtained the averaged concentration profiles across the slit, which are shown in Figure 1b–c. Since the slit walls were charged, an electric double layer was built at each wall; its structure and thickness are consistent with the results obtained earlier by a SPC/E model of water [35,36,37]. The density distribution of polymer beads is also in good agreement with the previous studies [38]. The solvent density shows an oscillatory behaviour at the walls (Figure 1c) and is reduced due to the presence of the polymers (Figure S1).

Our primary interest was to study the hydrodynamic properties of polymer coatings, screening the electro-osmotic flow. An important quantity characterizing such screening is the effective hydrodynamic (or Stokes) radius of the polymer bead, $a_{\mathrm{bead}}$, which appears in the continuous, one-dimensional (along the *z* axis, see Figure 1a) Navier-Stokes-Brinkmann (NSB) equation (see Equation (1)). In order to calculate $a_{\mathrm{bead}}$, we fitted the averaged solvent velocity profile across the slit, obtained from the MD simulations, to the velocity profile calculated from the NSB equation. For the parameters of the NSB equation (such as viscosity, density of beads, etc.), we used the values obtained directly from the MD simulations, so that the bead’s hydrodynamic radius was the only fitting parameter. Figure 1d demonstrates an excellent match between the MD and NSB approaches, and suggests that the continuous NSB model can describe well the electro-osmotic flow screened by polymers (for additional velocity profiles see Figure S2). A similar agreement between the two approaches has been reported by Raafatnia et al. [34]. It is important to point out that the flow is still in the linear regime even for relatively strong applied electric fields (the inset in Figure 1d).

### 3.1. The Stokes Radius of Beads in Polymeric Coatings

We next calculated the hydrodynamic radius of polymer beads for different degrees of polymerization *N* (in our simulations, a polymer of degree of polymerization *N* consisted of 3N+2 “free” beads, see Section 2). The range of *N* considered covers the mushroom and brush-like configurations, which correspond to the gyration radius smaller and larger than the polymer-polymer separation, respectively; for our set-up, the crossover between the two conformations occurs at N=14 (note that another possibility to obtain the transition between the brush and mushroom configurations is to fix the polymer length and to vary the grafting density).

Figure 1e demonstrates two important features of the hydrodynamic screening:

1. The hydrodynamic radius, $a_{\mathrm{bead}}$, is about one order of magnitude smaller than the physical (i.e., Lennard-Jones) radius of the polymer bead. This result is consistent with the previous studies [25,34], but its origin does not seem to be well understood.
2. The hydrodynamic radius decreases at a decreasing slope with increasing the degree of polymerization, *N*. This is in agreement with the decreasing center-line flow velocity (i.e., the solvent velocity measured in the middle of the slit). This result is a new finding and implies that $a_{\mathrm{bead}}$ depends on the configuration of polymers, as suggested by Monteferrante et al. [47].

We rationalized these findings by studying the structure and the hydrodynamic properties of polymeric coatings, as described in detail below.

### 3.2. Structure of Polymeric Coatings

The crucial assumption in the continuous NSB approach is that polymer beads are treated as isolated resistance centers screening the flow. However, the polymers are chains of connected beads and the screening properties of a bead shall be affected by the beads surrounding it; in other words, the effective hydrodynamic radius of the polymer beads is expected to depend on the structure of a polymer coating [47], which, in turn, depends on the polymer’s length and polymer-polymer separations.

**Figure 2:**
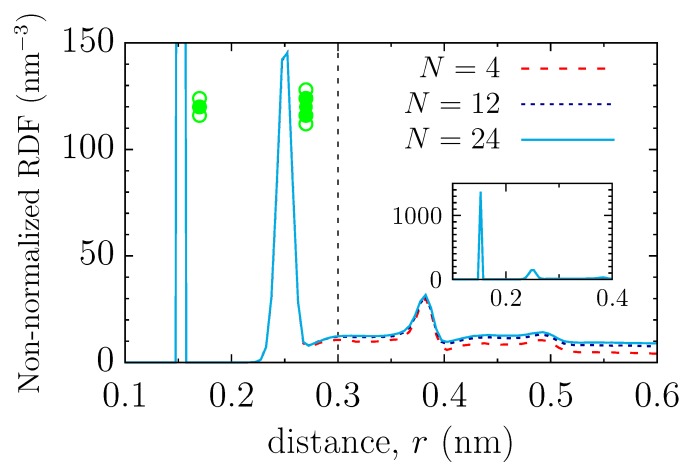
Structural properties of polymer coating. Non-normalized radial distribution function (RDF) is shown for a few polymers of different length *N*. The inset demonstrates a delta-like peak at r=0.15 nm, arising due to the two next-neighboring beads, as depicted in the sketch. The thin vertical line at $r_{0}=0.3$ nm separates the short and long distance regimes, where the effect of polymer length is negligible and appreciable, respectively. The empty circles in the sketches show the beads of a polymer that contribute the most to the corresponding peaks.

To reveal the conformational structure of the grafted polymers, we have calculated the gyration radius of polymers (Figure S3, Section S1 in Supplementary Materials) and the bead-bead radial distribution function (RDF, Figure 2; since the bead density in the system is essentially inhomogeneous, we have left the RDF non-normalized). The RDF unveils that the short-distance structure is practically independent of the polymer length (*N*). The first peak in the RDF results from two neighbouring beads, and the second peak from the next-nearest beads, of the same polymer. The noticeable dependence of the RDF on *N* is apparent only for distances larger than $r_{0}\approx 0.3$ nm, beyond which the polymer structure becomes influenced by the beads from the neighbouring polymers.

It is reasonable to expect that the hydrodynamic properties of polymer beads are different in these two regions, and we thus studied such properties separately for near and far beads.

### 3.3. Hydrodynamic Screening by Near Beads

**Figure 3:**
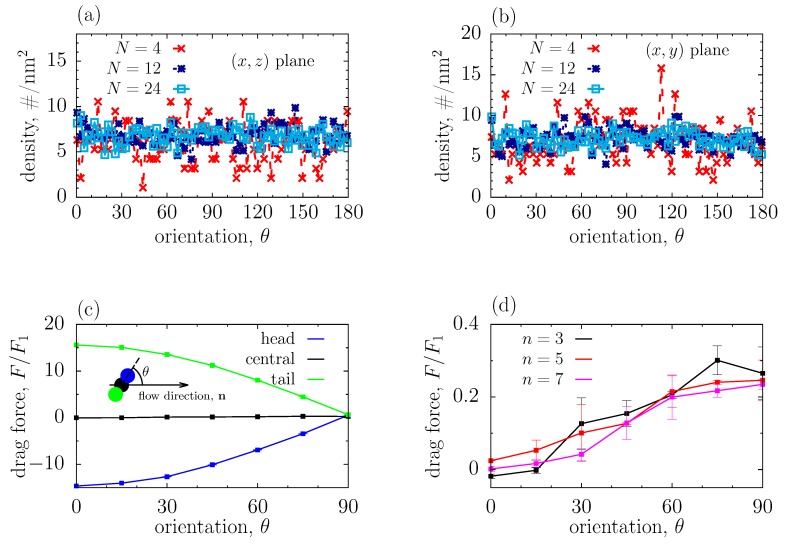
Near bead orientation and flow shielding. (**a**,**b**) Orientation distribution of the nearest bead in a polymeric coating within the (x,z) and (x,y) planes (see Figure 1a) for different degrees of polymerization; (**c**) Drag force acting on the beads in a three-bead configuration as a function of orientation in the (x,z) plane. The positions of all beads are fixed and the beads are not allowed to fluctuate. The drag force is expressed in terms of the drag acting on the bead in the absence of other beads, $F_{1}\approx 4.7$ kJ/(mol nm); (**d**) Drag force acting on the central bead for different numbers of beads in a sample.

To understand better the configuration of, and the hydrodynamic shielding by near beads, we first calculated the orientation distribution function, *f*, that is, the surface density of beads on a sphere of radius *r* as a function of orientation, θ. Figure 3a,b shows f(θ) at r=0.15 nm (the separation between two beads in a polymer) calculated in the (x,z) and (x,y) planes, i.e., in the planes perpendicular and parallel to the slit walls, respectively; here θ denotes the angle with respect to the direction of flow (the sketch in Figure 3c). This figure demonstrates that there is no preferred orientation of beads, independently of the degree of polymerization. This is likely because there is only weak or vanishing force from the electro-osmosis acting on the nearest beads, which might have caused a non-homogeneous distribution of these beads.

Having gained some insight into the near-bead structure, we decided to study the hydrodynamic shielding and its dependence on the bead orientation. For clarity, we focused on a simplified model, consisting of three aligned beads located in a channel under electro-osmotic flow. The beads were placed at a given angle with respect to the flow direction $n_{\mathrm{flow}}$ (the sketch in Figure 3c) and were not allowed to fluctuate. We took the bead separation the same as that in the polymer coatings (0.15 nm), and applied a strong electric field ($E_{x}=8\times 10^{-2}$ V/nm), in order to calculate the drag force acting on the beads with good accuracy. To reduce the hydrodynamic influence of the periodic images, we also extended the system in the flow direction to 19.76 nm.

Figure 3c shows the hydrodynamic drag acting on each bead as a function of orientation in the (x,z) plane. The forces acting on the head and tail beads are opposite in sign and depend vividly on θ; they are remarkably strong for the beads aligned along the flow, $n_{\mathrm{flow}}$ (corresponding to θ=0), but become orders of magnitude weaker for the beads oriented perpendicular to $n_{\mathrm{flow}}$ (corresponding to $\theta =90^{\circ }$). This is because, for θ≈0, the interaction of these beads with the solvent is limited due to screening by their neighbouring beads. Indeed, the tail bead, which faces the flow, interacts only with the solvent on its left side (see the sketch in Figure 3c), and hence there is a strong force, $F_{\mathrm{tail}}$, acting along the flow. In contrast, the head bead experiences only repulsive interactions with the solvent on its right side, which leads to a strong force pointing in the direction opposite to $n_{\mathrm{flow}}$ (this force is thus opposite in sign to $F_{\mathrm{tail}}$, and also slightly weaker, because the flow leads to the solvent density that is slightly lower in front of the head bead, as compared to the back of the tail bead (Figure S4). As the orientation changes to perpendicular, the beads interact with the solvent from *both* sides; this means that the forces acting on the left and right sides of these beads partially compensate each other, and the magnitude of the total force decreases drastically.

For the same reason, the hydrodynamic drag on the central bead, $F_{\mathrm{central}}$, is greatly reduced as compared to the head and tail beads; it is also much weaker than the drag acting on a single bead (i.e., with no other beads around). Figure 3d shows that $F_{\mathrm{central}}$ practically vanishes for θ=0, when it is determined mostly by the viscous sliding of the solvent. It increases roughly linearly with θ, achieving the maximum at $\theta =90^{\circ }$; in this case, $F_{\mathrm{central}}$ is mainly due to the normal stress exerted by the solvent, as discussed above. Interestingly, $F_{\mathrm{central}}$ is virtually independent of the number of beads in a sample, which suggests that the screening is predominantly due to the two nearest beads.

To estimate the value of the hydrodynamic radius due to shielding by near beads, we proceeded as follows. First, for a given orientation of just two near beads (angle θ, Figure 3c), we calculated the ambient velocity, *v*, at the same height (*z* coordinate) as the position of the central bead (it was calculated in the region sufficiently far from the beads, see Figure S4). Then, we used the Stokes law, which gives for the hydrodynamic radius $a_{\mathrm{bead}}\left(\theta \right)=F_{\mathrm{central}}\left(\theta \right)/\left[6\pi \mu v\left(\theta \right)\right]$, where μ is the viscosity. Figure 3a,b show that the distribution of near beads in the polymer coatings is homogeneous, i.e., the normalized distribution density is uniform; thus, in order to get a rough estimate of $a_{\mathrm{bead}}$, we averaged $a_{\mathrm{bead}}\left(\theta \right)$ over θ (Section S2 in Supplementary Materials), which gave $a_{\mathrm{bead}}\approx 0.18a_{0}$, where $a_{0}$ is the physical bead radius. This value is higher than $a_{\mathrm{bead}}$ obtained by using the continuous Navier-Stokes-Brinkmann (NSB) model for polymer coatings (Figure 1e). More importantly, such near-bead considerations do not bring any dependence on the degree of polymerization, in contrast to the results of the NSB model.

### 3.4. Shielding by Far Beads

**Figure 4:**
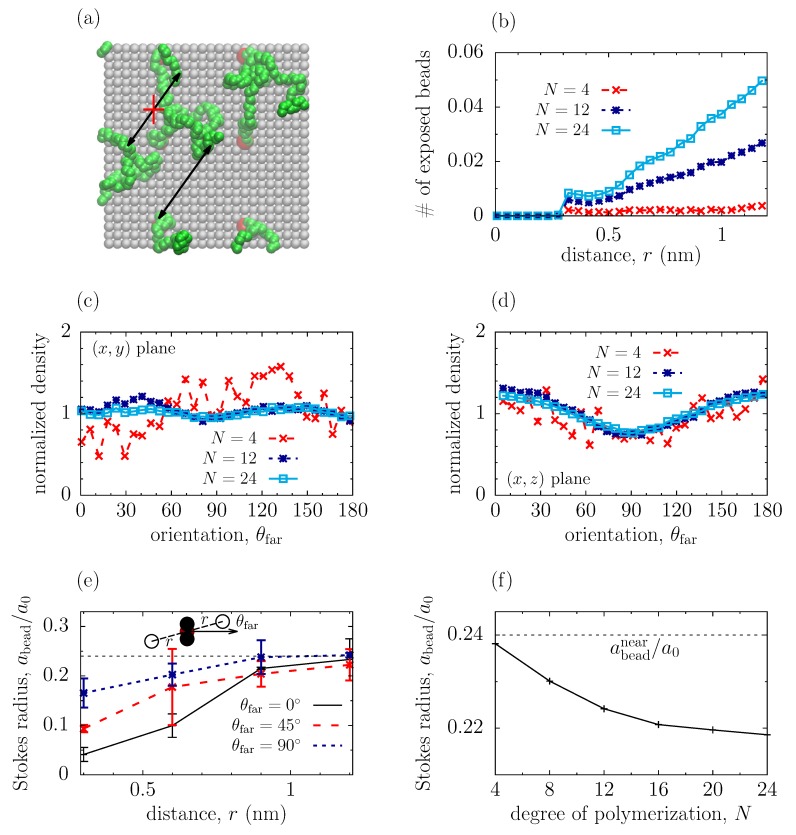
Far bead structure and flow shielding. (**a**) Snapshot from MD simulations showing the top view on the bottom surface. The wall atoms are shown in grey, the polymer beads in green, and the beads attached to the wall are shown in red. The arrows demonstrate the separations between far beads, and the crossed arrow demonstrates the separations not counted in the distribution function shown in panel (**b**); (**b**) Average number of far beads directly exposed to each other (see text) in the polymeric coating of Figure 1a as a function of separations between the beads; (**c**,**d**) Orientation distribution of far beads in the polymeric coating calculated in the (x,z) and (x,y) planes (see Figure 1a) for different degrees of polymerization, *N*. The density is normalized to the average density at given *N*; (**e**) Screening of the central bead of a three-bead complex by two far beads located symmetrically around the complex (see the sketch); (**f**) Hydrodynamic radius of the central bead of the complex as a function of the degree of polymerization calculated by using Equation (2). In (**e**,**f**), the horizontal dash lines denote the value of the hydrodynamic radius $a_{\mathrm{bead}}^{\mathrm{near}}$ due to shielding by near beads only; $a_{\mathrm{bead}}$ is expressed in terms of the physical radius $a_{0}=0.153$ nm. Compare with Figure 1e.

To rationalize the dependence of the effective Stokes radius on the degree of polymerization, we studied screening by far beads. It is reasonable to expect, however, that the contribution to screening from a far bead is small or negligible, if this bead itself is “screened” by other far beads. Thus, in our analysis, we considered only those beads that are directly “exposed” to each other, i.e., when there is no other bead between them (we took it, quite arbitrarily, to be within the bead diameter). Figure 4b shows the number of such beads, $n_{\mathrm{far}}\left(r\right)$, with one bead being at a distance z=1 nm from the bottom wall (see Figure S5 for the distribution of far beads at z=2 nm). As one may expect, $n_{\mathrm{far}}$ increases with the degree of polymerization *N*. Similarly as for the near beads, the distribution of far beads is practically independent of their orientation (Figure 4c,d); here, however, some deviations are visible in the (x,z) plane, but their magnitude is relatively small.

To understand the hydrodynamic screening by far beads, we considered first a simplified model system, consisting of a three-bead complex with two “far” beads placed symmetrically at a distance *r* from the complex and at an angle $\theta_{\mathrm{far}}$ with respect to the direction of flow (the sketch in Figure 4e); as before, the flow was induced by applying an electric field $E_{x}=8\times 10^{-2}$ V/nm in the *x* direction. The placement of far beads was chosen such as to avoid complications with balancing collisions of the solvent from both sides of the complex. All far and near beads in this system were frozen and disallowed to fluctuate. We then calculated the hydrodynamic radius of the *central bead* in the complex as $a_{\mathrm{bead}}\left(\theta_{\mathrm{far}}\right)=F\left(\theta_{\mathrm{far}}\right)/\left[6\pi \mu v\left(\theta_{\mathrm{far}}\right)\right]$, where *F* is the drag acting on the central bead, *v* is the ambient velocity at a given *z* position and μ the viscosity. The result is shown in Figure 4e, which demonstrates that $a_{\mathrm{bead}}$ increases with increasing *r* and saturates at $a_{\mathrm{bead}}^{\mathrm{near}}$, where $a_{\mathrm{bead}}^{\mathrm{near}}$ is the hydrodynamic radius due to screening by near beads only (in our case, the near beads were oriented perpendicular to the flow, i.e., $\theta =90^{\circ }$, see Figure 3c). We shall call a shielding radius (or shell), $r_{s}$, a distance at which the effect of far beads becomes negligible, i.e., at which $a_{\mathrm{bead}}\left(\theta_{\mathrm{far}}\right)\approx a_{\mathrm{bead}}^{\mathrm{near}}$. As one may expect, the effect of far beads becomes weaker as they are rotated to be perpendicular to the flow ($\theta_{\mathrm{far}}=90^{\circ }$), and the shielding radius decreases correspondingly.

To relate the structural information about the polymer coatings (Figure 4a–d) with their hydrodynamic properties, we proceeded as follows. We assumed that the far-bead screening is additive and ignored collective effects, which allowed us to average the hydrodynamic radius over the orientation and spatial distribution of far beads in a polymer coating. Splitting this average into the regions within and outside of the screening shell, and formally extending the integration over *r* to infinity for generality, we have arrived at (Section S3 in Supplementary Materials).

(2) $$\begin{array}{c} a_{\mathrm{bead}}\left(N\right)=a_{\mathrm{bead}}^{\mathrm{near}}-\int_{0}^{\pi /2}d\theta_{\mathrm{far}}\int_{0}^{\infty }dr\left[a_{\mathrm{bead}}^{\mathrm{near}}-a_{\mathrm{bead}}\left(r,\theta_{\mathrm{far}}\right)\right]P_{\mathrm{far}}\left(r,\theta_{\mathrm{far}};N\right), \end{array}$$

where $P_{\mathrm{far}}\left(r,\theta ;N\right)=\left(1/2\right)n_{\mathrm{far}}\left(r;N\right)\sin\left(\theta \right)$ is the distribution function of far beads (Section S2 in Supplementary Materials), assuming that the orientation distribution of beads is homogeneous (Figure 4c,d).

Figure 4f shows the dependence of $a_{\mathrm{bead}}$ on *N* obtained by using Equation (2). Although the predicted $a_{\mathrm{bead}}$ differs from the $a_{\mathrm{bead}}$ obtained by using the NSB equation (Figure 1e), the qualitative behaviour is similar. The hydrodynamic radius is further reduced by far beads and decreases as *N* increases. This decrease is stronger in the mushroom region (N<14; similarly as in Figure 1e), where the number of far beads rises quickly with increasing *N*.

The discrepancies with the NSB model can be due to a number of reasons. Firstly, to demonstrate the effect of far beads, $a_{\mathrm{bead}}$ in Figure 4 has been calculated only for a near-bead complex oriented perpendicular to the flow ($\theta =90^{\circ }$). Averaging over θ shall further reduce $a_{\mathrm{bead}}$, bringing it closer to the NSB values (this has not been done due to computational limitations). Further, we have neglected the collective effects and the contribution from ‘partially exposed’ beads and bead complexes, which are likely to reduce $a_{\mathrm{bead}}$ as well. It will be interesting to account for these effects in future work.

## 4. Conclusions

We have studied the hydrodynamic properties of polymeric coatings by using molecular dynamics (MD) simulations and the continuum Navier-Stokes-Brinkman (NSB) model. By matching the velocity profiles from the MD and NSB approaches, we inferred the effective hydrodynamic radius of polymer beads, $a_{\mathrm{bead}}$. The obtained $a_{\mathrm{bead}}$ is about one order of magnitude smaller than its physical (Lennard-Jones) radius, in agreement with the previous studies [25,34]. Furthermore, we showed that $a_{\mathrm{bead}}$ decreases with increasing the degree of polymerization of coated polymers at a decreasing slope (Figure 1e).

We rationalized the reduced hydrodynamic radius by linking the structural and hydrodynamic properties of polymer coatings. We observed that, at short distances, the polymer structure is unaffected by the polymer length (Figure 2). In this case, by studying the hydrodynamic shielding by near beads explicitly by MD simulations, we demonstrated that two near beads contribute the most to the reduction of the hydrodynamic radius (Figure 3d). Using the distribution function of a polymer coating and the hydrodynamic information from the near-bead model, both obtained by MD simulations, we found that the effective hydrodynamic radius due to the near-bead shielding is $a_{\mathrm{bead}}^{\mathrm{near}}\approx 0.18a_{0}$, where $a_{0}$ is the physical (Lennard-Jones) radius, and is independent of the polymer length or grafting density.

The hydrodynamic shielding by far beads turned out to be more intricate. Using MD simulations, we showed that the effective hydrodynamic radius is practically unaffected by far beads if they are beyond a ‘shielding radius’ (which depends on the orientation and size of a screening object; Figure 4e). Making use of this fact, and using a number of simplifying assumptions, we suggested a simple model relating the structural properties of polymer coatings and the hydrodynamics of far-bead screening. This model predicts a further reduction of the effective hydrodynamic radius and brings about its dependence on the degree of polymerization *N* (Figure 4f). Although the resulting hydrodynamic radius is sill larger than the one obtained by the NSB model, its dependence on *N* seems to be captured properly.

The reported shielding effects may have a broad impact on the understanding of the hydrodynamic properties of polymer coatings. Indeed, we demonstrated a subtle correlation between the hydrodynamic and structural properties, particularly that the hydrodynamic radius is larger in the mushroom region and smaller for the brush configurations of polymer coatings. It has been shown by Hill [25] that the bead’s hydrodynamic radius of *charged* polymers is three times larger than that of neutral polymers. Our work suggests that this might in fact be related to different structures adapted by these polymers, in particular that the polyelectrolytes prefer ‘stretched-up’ configurations [13], in contrast to neutral polymers. Thus, it may be interesting to explore further such hydrodynamics-structure relations.

## Supplementary Materials

The following are available online at https://www.mdpi.com/2073-4360/11/6/1038/s1, Figure S1: Solvent density of systems with and without polymers, Figure S2: Velocity profiles from the MD simulations and the NSB model, Figure S3: Gyration radius of polymers, Figure S4: Velocity and density distributions around bead complex, Figure S5: Distribution of far beads around central bead, Figure S6: Fluid viscosity, Section S1: Gyration radius of polymers, Section S2: Averaging over bead orientations, Section S3: Averaging over far beads, Section S4: Navier-Stokes-Brinkman model, Table S1: Parameters of molecular dynamics simulations, Table S2: Force field parameters, grompp.mdp, poly.gro, poly.itp, force.itp, top.top: Parameter, coordinate and topology files of MD simulations.
