# Supplementary material for: Hydrodynamic Properties of Polymers Screening the Electrokinetic Flow: Insights from a Computational Study
Source: Polymers (Basel). 2019 Jun 11;11(6):1038. doi: 10.3390/polym11061038 (PMC6631430; doi:10.3390/polym11061038)
Supplement: Supplementary file 1 [file polymers-11-01038-s001.zip › Supplementary_materials/ssek-si.pdf]

Supplementary Materials

Hydrodynamic Properties of Polymers Screening the Electrokinetic Flow: Insights from a Computational Study

Peng Wu, Sun Tao, Xikai Jiang, and Svyatoslav Kondrat

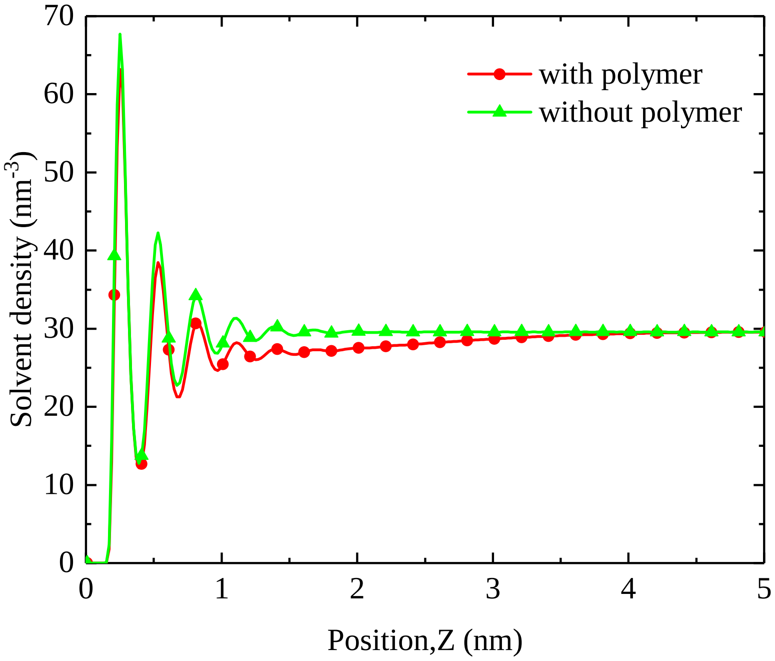

FIG. S1. **Solvent density of systems with and without polymers.** With attached polymers, the solvent density is reduced, as compared to the density of the system without polymers, but the bulk value is the same in both systems. The parameters are the same as in Fig. 1 of the main text.

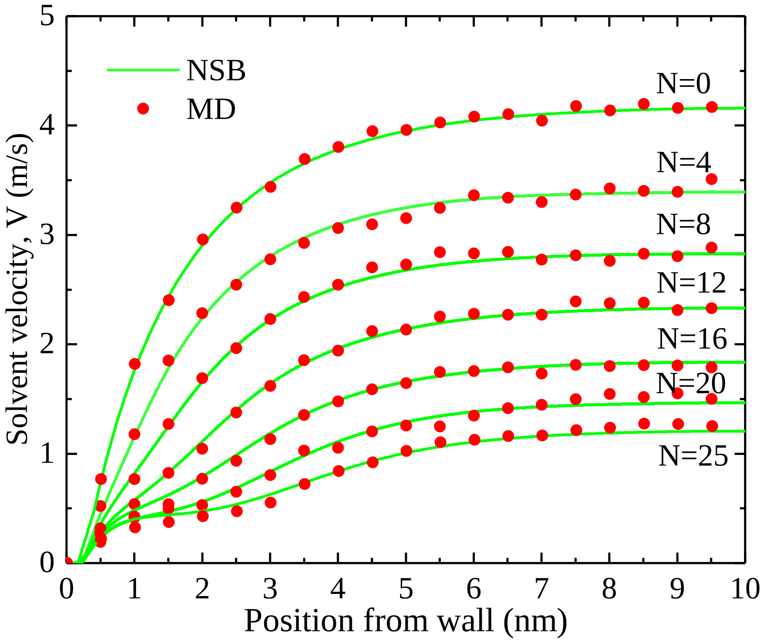

FIG. S2. **Velocity profiles from the MD simulations and the NSB model.** The velocity profiles are shown for a few polymer lengths, N. The agreement between the two approaches is reasonably good for all values of N.

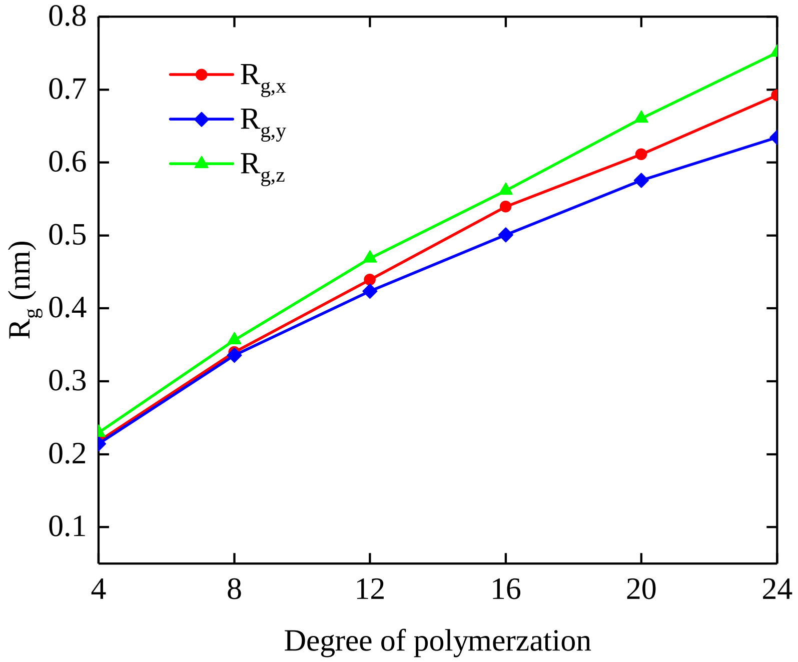

FIG. S3. **Gyration radius of polymers in parallel (x, y) and perpendicular (z) directions with respect to wall.**

S1. GYRATION RADIUS OF POLYMERS

The gyration radius of polymers along axis α is [1]

$$R_{g,\alpha} = \sqrt{\frac{1}{N}\langle\sum_{i=1}^{N}[r_{\alpha,i} - \bar{r}_{\alpha}]^2\rangle}, \tag{S1}$$

where the coordinates of the ith bead are $(r_{x,i}, r_{y,i}, r_{z,i})$, the center of mass of polymers $\bar{r} = (r_x, r_y, r_z)$, and $\langle \cdots \rangle$ denotes the statistical average. The result (Fig. S3) shows that the variation of gyration radius is different in parallel (x, y) and perpendicular (z) directions. Specially, the size of a polymer is larger in the perpendicular direction and increases with increasing the degree of polymerization. This is likely because the configuration of polymer changes from the mushroom to the brush-like shapes. In addition, the parallel size of polymer in the x direction is larger than that in the y direction. This phenomena might originate from the deformation of polymers induced by the flow along the x direction.

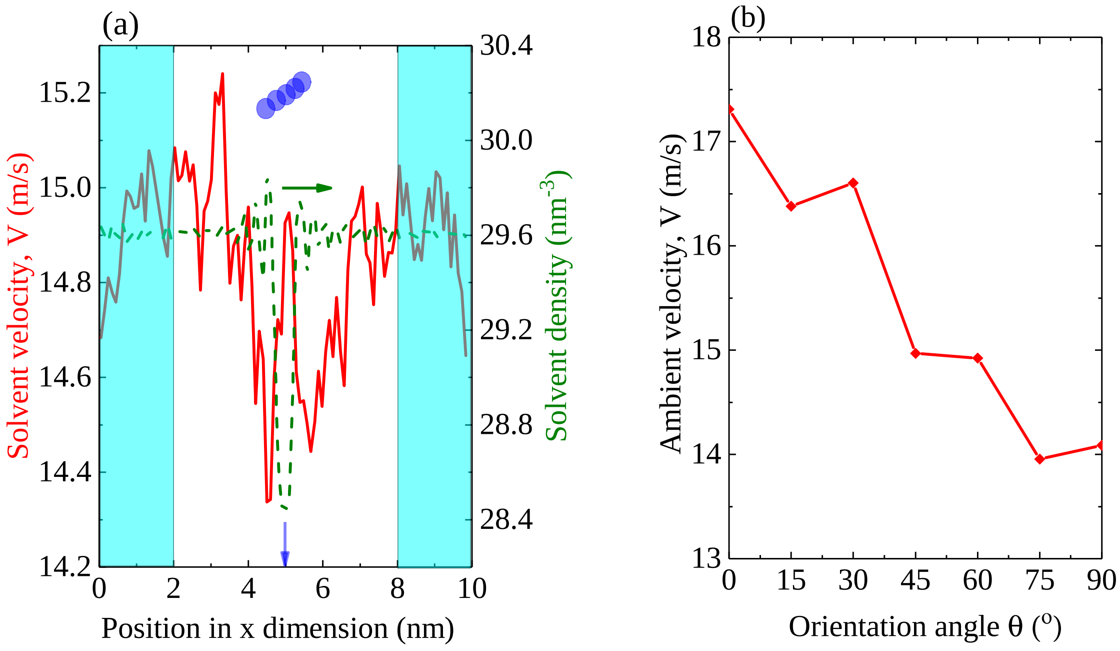

FIG. S4. **Velocity and density distributions around a bead complex.** (a) Distribution of solvent velocity (red) and solvent density (green) for the bead complex oriented at 45° to the flow direction. The ambient velocity was calculated in the blue shaded area. (b) Ambient velocity as a function of orientation of the bead complex (see the inset in Fig. 3c of the main text). This velocity has been used in the Stokes equation to obtain the hydrodynamic radius due to the near bead shielding.

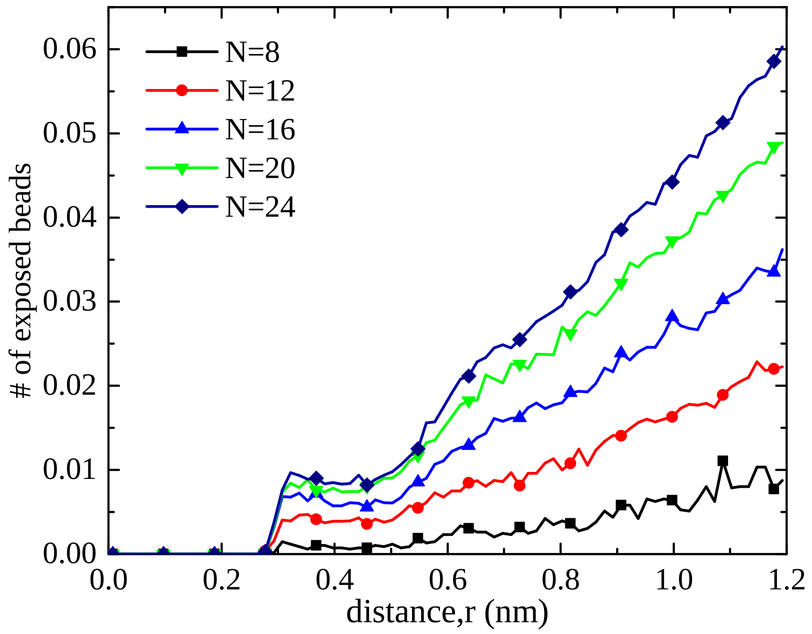

FIG. S5. **Distribution of far beads around central beads located at $z = 2$ nm away from the wall.** Although the concentrations of beads are slightly different, as compared to the distribution with $z = 1$nm (Fig. 4b of the main text), the general features remain the same, in particular that the number of beads increases with N, and that their relative increase slows down as N increases. Distribution of far beads for $N = 4$ mer is not shown because the height of such polymer is lower than 2 nm.

S2. AVERAGING OVER BEAD ORIENTATIONS

For bead configurations as in the insets of Fig. 3c and 4e (of the main text), the number of beads at position r within a spherical element $d\theta d\phi$ is $\rho r^2 \sin(\theta) d\theta d\phi$, where $\rho(r, \theta, \phi)$ is the surface density of beads at distance r and orientation (θ, ϕ), with θ and ϕ being the polar and azimuthal angles, respectively. Assuming that the amount of beads varies little with the orientation, *i.e.*, $\rho \approx \rho(r)$ (Fig. 3a-b and Fig. 4c-d of the main text), and taking into account that configurations (θ, ϕ) and $(\pi - \theta, \pi - \phi)$ are equivalent for both near and far bead configurations (Fig. 3c and Fig. 4e of the main text), the *orientation* averaging of $a(r, \theta)$ is

$$\langle a \rangle_\theta(r) = \int_0^{\pi/2} a(\theta) P(r, \theta) d\theta, \tag{S2}$$

where

$$P(r, \theta) = 2\pi \rho(r) r^2 \sin(\theta), \tag{S3}$$

and we have used the ϕ-independence of a.

To calculate the averaged hydrodynamic radius due to the near-bead shielding, we took $\rho \approx 7.07\ \mathrm{nm}^{-2}$ (see Fig. 3a-b of the main text) and $r = r_{\mathrm{near}} = 0.15\mathrm{nm}$, which corresponds to the separation between two neighbouring beads in a polymer. Note that $2\pi\rho(r_{\mathrm{near}}) r_{\mathrm{near}}^2 \approx 1$.

For far beads, from molecular dynamics simulations, we calculated the average number of beads at position r, $n_{\mathrm{far}}(r)$ (Fig. 4b of the main text). Assuming again the orientation independence of the far bead distribution, n_{far} is

$$n_{\mathrm{far}}(r) = 4\pi r^2 \rho(r). \tag{S4}$$

Using eqn (S3), the far-bead distribution function becomes (see eqn (1) in the main text)

$$P_{\mathrm{far}}(r, \theta) = \frac{1}{2} n_{\mathrm{far}}(r) \sin(\theta). \tag{S5}$$

S3. AVERAGING OVER FAR BEADS

Let us consider a bead (say, bead 1) and observe the behaviour of another bead (bead 2) with respect to bead 1. In the course of a simulation, we can split the simulation time T into time T_0, when bead 2 is beyond the shielding radius r_0, and times $T(r)$ when it is within r_0 at distance r. Then, for an observable with the property that $a = a_0$ outside the shielding shell and $a = a(r)$ for $r < r_0$, the average is

$$\bar{a} = \frac{a_0 T_0}{T} + \frac{1}{T} \int_{r<r_0} a(r) T(r). \tag{S6}$$

Using the obvious identity $T = T_0 + \int_{r<r_0} T(r)$, we obtained

$$\bar{a} = a_0 - \int_{r<r_0} P(r)[a_0 - a(r)], \tag{S7}$$

where $P(r) = T(r)/T$ is the probability of bead 2 to be distance r away from bead 1. Assuming the additivity of far bead screening, we performed the average over all polymer beads in a sample, which yields

$$\bar{a} = a_0 - \int_0^{\pi/2} d\theta \int_0^{r_0} dr P(r,\theta)[a_0 - a(r)], \tag{S8}$$

where $P(r,\theta)$ is given by eqn (S5).

S4. NAVIER-STOKES-BRINKMAN MODEL

The Navier-Stokes-Brinkman (NSB) model is a continuous model, in which the velocity of an electro-osmotic flow is determined by the one-dimensional Navier-Stokes equation, supplemented by the Brinkman term, describing the effect of polymers [2]. The NSB equation is given by eqn (2) in the main text. This equation was used to extract the effective Stokes radius of polymer beads. The computational details are provided below.

A. Drag coefficient

The NSB model requires the drag coefficient $K(\phi)$, where $\phi(z)$ is the bead volume fraction (which depends on the position z across the wall). We have used the expression provided by Koch and Sangani [3] (and also used by Hill [2]), which reads:

$$K(\phi) = \frac{1 + 3[\phi/2]^{1/2} + (135/64)\phi \ln \phi + 16.456\phi}{1 + 0.681\phi - 8.48\phi^2 + 8.16\phi^3}. \tag{S9}$$

B. Fluid viscosity

To calculate the viscosity for the system with polymer-grafted walls, we first used molecular dynamics (MD) simulations to determine the *bulk* viscosity, μ_0, of the system *without* polymers. The applied electric field was $E_x = 8 \times 10^{-2}$ V/nm; we checked that the obtained viscosity is valid in the range of E_x used in this work (from 1.6×10^{-2} V/nm to 1.6×10^{-1} V/nm).

In principle, the viscosity of the system without polymers, μ_0, depends on the position across the slit. A useful method to compute the position-dependent viscosity has been proposed by Todd *et al.* [4]. For simplicity, however, we use here a constant (bulk) viscosity for μ_0, which seems sufficient for our purposes. We have checked, by using the method of Ref. [5], that accounting for the enhancement in μ_0 in the near-surface layer, does not change our results qualitatively.

To take into account the presence of polymers, we note that the polymer bead density in our simulations was low (about 5 beads per nm^3, see Fig. 1c in the main text). This allows the application of the low density expansion and the Einstein relation model [6], which

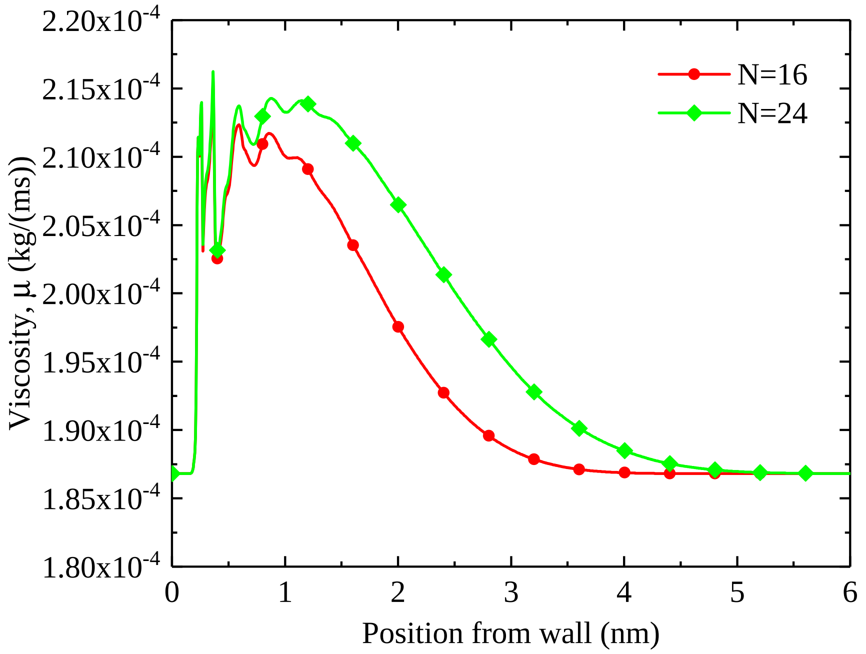

FIG. S6. **Fluid viscosity.** The viscosity of a fluid as determined by eqn (S10) for two values of the degree of polymerization $N = 16, 24$.

describes the increase of viscosity due to the suspension of polymer beads. This model gives

$$\mu(z) = \mu_0[1 + 2.5\phi(z)], \tag{S10}$$

where $\phi(z)$ is the volume fraction of polymer beads, which we extracted directly from the MD simulations. Two examples of the so-calculated viscosity are shown in Fig. S6.

C. Boundary conditions for the NSB model

Our molecular dynamics simulations show that the solvent velocity vanishes at $z_0 \approx 0.17$ nm (Fig. S2). Thus, in the NSB model we applied the non-slip boundary condition at $z = z_0$, *i.e.*,

$$u\,|_{z=z_0} = 0. \tag{S11}$$

We also made use of the mirror symmetry with respect to the mid-plane ($z = w/2$), which amounts to setting

$$\left.\frac{du}{dz}\right|_{z=w/2} = 0, \tag{S12}$$

where w is the slit width.

REFERENCES

[1] M. Jehser, G. Zifferer, and C. N. Likos, "Scaling and interactions of linear and ring polymer brushes via dpd simulations," Polymers **11**, 541 (2019).

[2] R. J. Hill, "Hydrodynamics and electrokinetics of spherical liposomes with coatings of terminally anchored poly(ethylene glycol): numerically exact electrokinetics with self-consistent mean-field polymer," Phys. Rev. E. **70**, 051406 (2004).

[3] D. L. Koch and A. S. Sangani, "Particle pressure and marginal stability limits for a homogeneous monodisperse gas-fluidized bed: kinetic theory and numerical simulations," J. Fluid Mech. **400**, 229–263 (1999).

[4] B. D. Todd, Denis J. Evans, and Peter J. Daivis, "Pressure tensor for inhomogeneous fluids," Phys. Rev. E **52**, 1627–1638 (1995).

[5] P. Wu and R. Qiao, "Physical origins of apparently enhanced viscosity of interfacial fluids in electrokinetic transport," Phys. Fluids **23**, 072005 (2011).

[6] G. K. Batchelor and J. T. Green, "The determination of the bulk stress in a suspension of spherical particles to order c2," J. Fluid Mech. **56**, 401427 (1972).

TABLE S1. Parameters of molecular dynamics simulations.

Parameters	Symbol	Value	Unit
Lateral dimension of channel	L_x	9.88,19.76	nm
Lateral dimension of channel	L_y	9.88	nm
Height of channel	H	20	nm
Density of wall atoms	ρ_w	33.3	nm^{-3}
Surface charge density	σ_s	3.28×10^{-2}	$\mathrm{C/m}^2$
Grafting density of polymers	σ_p	0.164	nm^{-2}
Degree of polymerization	N	4,8,12,16,20,24	N/A
Density of solvent	ρ_s	49.1	mol/L
Ionic strength of electrolyte	I	3.4×10^{-2}	mol/L
Strength of external electric field	E_{ext}	1.6×10^{-2},8×10^{-2}	V/nm
Solvent dielectric constant	ε_s	78	N/A
Viscosity of bulk solvent	μ_s	1.868×10^{-4}	$\mathrm{kg\,m^{-1}\,s^{-1}}$
Temperature of system	T	300	K
Pressure of system	P	1	bar
Time step of MD system	ΔT	4	fs

TABLE S2. Force field parameters for the ions, solvent and polymer interactions.

nonbonded parameters			
Atom type	Charge	σ (nm)	ϵ (kJ/mol)
Solvent	0	0.3	2.49
Na	+1	0.3	2.49
Cl	-1	0.3	2.49
Polymer beads[a]	0	0.3	2.49
Wall atoms[b]	some charge[c]	0.3	2.49

parameters for Lennard-Jones interaction for interaction pairs		
Pair	σ (nm)	ϵ (kJ/mol)
Solvent-Na	0.21	2.49
Solvent-Cl	0.21	2.49
Solvent-Beads	0.3	2.49
Na-Beads	0.3	2.49
Cl-Beads	0.3	2.49
Wall-Beads	0.3	2.49
Solvent-Wall	0.3	2.49
Beads-Wall	0.3	2.49
Na-Wall	0.3	2.49
Cl-Wall	0.3	2.49

bond interaction of polymers		
bond	b_0 (nm)	k_b (kJ mol^{-1}nm^{-2})
Beads-beads	0.153	334720

angle parameter of polymers		
angle	θ_0 (°)	k_θ (kJ mol^{-1}rad^{-2})
Beads-beads	110.0	460.0

torsion coefficients for Rychaert-Bellemans functions (kJ mol^{-1})						
torsion	C0	C1	C2	C3	C4	C5
Beads-beads-beads-beads	1.8958	1.4930	-2.1667	0.5555	4.3333	-6.1111

[a] Polymer beads are denoted as beads in later.

[b] Wall atoms are denoted as wall in later.

[c] Wall atoms adjacent to electrolytes carry charges to produce surface charge density of 3.28×10^{-2} C/m^2.
